# *catena*-Poly[calcium-bis­[μ-*N*-(dimethyl­phosphino­yl)benzene­sulfonamidato]]

**DOI:** 10.1107/S1600536809032875

**Published:** 2009-09-19

**Authors:** Elizaveta A. Trush, Victor A. Trush, Tetyana Yu. Sliva, Irina S. Konovalova, Volodymyr M. Amirkhanov

**Affiliations:** a National Taras Shevchenko University, Department of Chemistry, Volodymyrska str. 64, 01033 Kyiv, Ukraine; b STC "Institute for Single Crystals", 60 Lenina ave., Khar’kov 61001, Ukraine

## Abstract

The crystal structure of the title calcium complex, [Ca(C_8_H_11_NO_5_PS)_2_]_*n*_, is composed of a polymeric chain, which is formed due to two bridging sulfonyl groups linking Ca^II^ ions in a O—S—O—Ca manner. Thus, the coordination environment of the Ca^II^ ions is composed of six O atoms belonging to the phosphoryl and sulfonyl groups of two chelate rings and two additional O atoms of two bridging sulfonyl groups. The coordination polyhedron of the central atom (2 symmetry) has a distorted octa­hedral geometry.

## Related literature

For general background see: Wojtczak *et al.* (1996 ▶); Purdy *et al.* (1989 ▶); Oehr & Suhr (1988 ▶); Berry *et al.* (1988 ▶); Pietraszkiewicz *et al.* (2002 ▶); Anand (1996 ▶); Shannon (1976 ▶). For the synthesis of the ligand, see: Kirsanov (1952 ▶); Kirsanov & Shevchenko (1954 ▶). For theoretical S—O distances in the free non-coord­inated ligand, see: Moroz *et al.* (2009 ▶). 
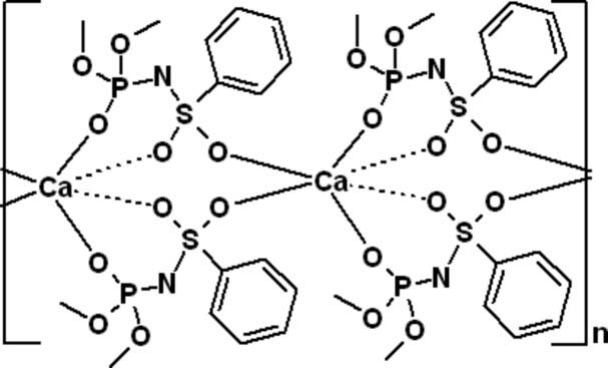


## Experimental

### 

#### Crystal data

[Ca(C_8_H_11_NO_5_PS)_2_]
*M* *_r_* = 568.50
Monoclinic, 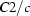

*a* = 20.692 (2) Å
*b* = 5.675 (1) Å
*c* = 22.178 (3) Å
β = 115.26 (1)°
*V* = 2355.3 (6) Å^3^
*Z* = 4
Mo *K*α radiation
μ = 0.63 mm^−1^
*T* = 293 K
0.40 × 0.20 × 0.10 mm

#### Data collection

Xcalibur’3 diffractometer
Absorption correction: multi-scan (*CrysAlis RED*; Oxford Diffraction, 2006 ▶) *T*_min_ = 0.785, *T*_max_ = 0.939
8999 measured reflections
3280 independent reflections
2503 reflections with *I* > 2σ(*I*)
*R*_int_ = 0.036

#### Refinement

*R*[*F* ^2^ > 2σ(*F* ^2^)] = 0.046
*wR*(*F* ^2^) = 0.139
*S* = 1.10
3280 reflections
152 parameters
H-atom parameters constrained
Δρ_max_ = 1.28 e Å^−3^
Δρ_min_ = −0.59 e Å^−3^

### 

Data collection: *CrysAlis CCD* (Oxford Diffraction, 2006 ▶); cell refinement: *CrysAlis RED* (Oxford Diffraction, 2006 ▶); data reduction: *CrysAlis RED*; program(s) used to solve structure: *SHELXTL* (Sheldrick, 2008 ▶); program(s) used to refine structure: *SHELXTL*; molecular graphics: *ORTEP-3 for Windows* (Farrugia, 1997 ▶); software used to prepare material for publication: *WinGX* (Farrugia, 1999 ▶).

## Supplementary Material

Crystal structure: contains datablocks I, global. DOI: 10.1107/S1600536809032875/bq2148sup1.cif

Structure factors: contains datablocks I. DOI: 10.1107/S1600536809032875/bq2148Isup2.hkl

Additional supplementary materials:  crystallographic information; 3D view; checkCIF report

## Figures and Tables

**Table:** 

Ca1—O3	2.3055 (15)
Ca1—O2	2.3392 (15)
Ca1—O1	2.3802 (14)

**Table:** 

O3^i^—Ca1—O3	152.17 (8)
O3—Ca1—O1	81.97 (6)
O2^i^—Ca1—O1	177.86 (5)

Symmetry code: (i) 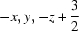.

**Fig. 1.**
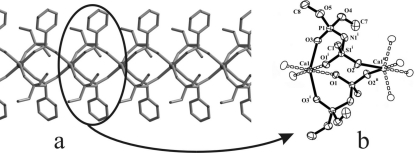
The polymeric chain of [Ca(sp)2], showing the formation of the cyclic motif (a); A view of [Ca(sp)2] (b) with displacement ellipsoids shown at the 30% probability level. H atoms and C6H5 groups have been omitted for clarity. Symmetry codes: (i) -x, y, -z + 3/2; (ii) x, y + 1, z.

## supplementary crystallographic information

### Comment

The coordination compounds of Group 2 metals with *O,O* - donor ligands have attracted much recent interest as potential precursors for the deposition of a range of electro-ceramic oxides by metal-organic chemical vapour deposition (MOCVD) (Wojtczak *et al.*, 1996; Purdy *et al.*, 1989). Various β- diketonate complexes of Group 2 have been proposed to preparation of deposit films of metals and metal oxides (Oehr *et al.*, 1988; Berry *et al.*, 1988).

N - Phosphorylated sulfonylamides (PS) of a general formula RSO_2_NHPO(*R*')_2_ can be considered as SNP - heterosubstituted structural analogues of β- diketones (HAD). They may be regarded as powerful chelating systems for various metal ions and coordination chemistry of HAD remains to be relatively wide elaborated (Pietraszkiewicz *et al.*, 2002).

The sulfonylamido-group –SO_2_NH– has been found as a key structural motif shared by a large number of bioactive compounds, spanning a wide variety of biological effects, such as antimicrobial activity, specific enzyme inhibition, hormone regulation, and among others used as chemotherapics (Anand, 1996).

The phosphorylic group in PS ligands possess high nucleophilic affinity. It can be used for obtaining of stable molecular or ionic [*M*(*L*)_2_]^-^, [*M*(*L*)_2_]^0^ and [*M*(*L*)_2_]^+^ species based on s-, *p*- and d-elements. Herein we report the synthesis and X-ray studies of coordination compound Ca(II) with dimethyl(phenylsulfonyl)amidophosphate.

There are no close contacts between the neighboring chains in the crystal (Fig. 1). The molecular species [Ca(*sp*)_2_] forms a polymer around the symmetry centre at *x*,*y*, *z* + 3/2 on calcium atom which is situated in the special position; Ca is immersed in a six-coordination environment provided by two bidentate *sp*- anion through sulfonyl O1 and phosphoryl O3 O atoms, and O2 of sulfonyl group connected in the bridge manner to the neighboring Ca atom. The resulting coordination polyhedron has the shape of slightly distorted octahedron with phosphoryl oxygen atoms {O3 and O3^i^} occupying the axial vertexes. The values of some bond lengths and angles are given in the Table 1. The angle O3 Ca1 O3^i^ has 152.17 (8)° and the corresponding distance Ca1 - O3 is 2.3055 (15) Å. [c.f. the sum of effective ionic radii are *M*^2+^+O^2-^(Ca, O)=2.35 Å] (Shannon, 1976); the mean deviation from plane O1O2O1^i^O2^i^Ca1 does not exceed 0.0157 Å only for central atom.

The chelating frame O1S1N1P1^i^O3^i^ is almost flat: the average deviation for all these atoms does not exceed 0.11Å with the maximum deviation recorded for P1^i^ (0.21 Å). In spite of this, the chelated metallo-cycle as a whole has an anomalous configuration: the value of angle between chelate and the plane Ca1O1O3^i^ is 158.5°.

SO_2_ group implementing the bridge function links the two calciums in a different manner (Scheme 1.).

Moreover, the distance metal-oxygen, implicated in metal-chelate somewhat longer than intermolecular bond Ca - O, which can be explained in the terms of packing effects.

This fact, in turn, did not lead to the noticeable appropriate increase in the length of SO (1.4547 (15) Å) and 1.4603 (14) Å) contacts (as always in the complex under coordination) in the comparison with theoretically identical distances in free noncoordinated ligand (Moroz *et al.*, 2009). Another bite bond lengths and angles around the atoms of phosphorus, nitrogen and sulfur have typical values for the appropriate substituted amidophosphates and sulfamides.

### Experimental

The synthesis of H(*sp*) was carried out according to previously reported method (Kirsanov, 1952; Kirsanov *et al.*, 1954). Calcium (0,01 g, 0,25 mmol) was dissolved in 5 ml of hot methanol and combined with a hot solution H(*sp*) (0,133 g, 0,5 mmol) in metanol (5 ml). The mixture was heated to 330 K for about 30 min. Colourless crystals of complex suitable for X-ray diffraction separated over a period of 7 days; they were washed with dry propan-2-ol and dried *in vacuo* at room temperature (0,134 g, 94%). Analysis found: IR (KBr pellet, cm^-1^): 1220, 1060 (s, SO_2_) and 1190 (s, PO).

### Refinement

All hydrogen atoms were located from electron density difference maps and included in the refinement in the riding motion approximation with *U*_iso_ constrained to be 1.5 times *U*_eq_ of the carrier atom for the methyl groups and 1.2 times *U*_eq_ of the carrier atom for the other atoms.

### Crystal data

**Table:**

[Ca(C_8_H_11_NO_5_PS)_2_]	*F*(000) = 1176
*M**_r_* = 568.50	*D*_x_ = 1.603 Mg m^−^^3^
Monoclinic, *C*2/*c*	Mo *K*α radiation, λ = 0.71073 Å
Hall symbol: -C 2yc	Cell parameters from 6842 reflections
*a* = 20.692 (2) Å	θ = 3.6–27.5°
*b* = 5.675 (1) Å	µ = 0.63 mm^−^^1^
*c* = 22.178 (3) Å	*T* = 293 K
β = 115.26 (1)°	Block, colourless
*V* = 2355.3 (6) Å^3^	0.40 × 0.20 × 0.10 mm
*Z* = 4	

### Data collection

**Table:**

Xcalibur'3 diffractometer	3280 independent reflections
Radiation source: Enhance (Mo) X-ray Source	2503 reflections with *I* > 2σ(*I*)
graphite	*R*_int_ = 0.036
Detector resolution: 16.1827 pixels mm^-1^	θ_max_ = 30.0°, θ_min_ = 3.6°
ω–scans	*h* = −25→29
Absorption correction: multi-scan (*CrysAlis RED*; Oxford Diffraction, 2006)	*k* = −7→7
*T*_min_ = 0.785, *T*_max_ = 0.939	*l* = −30→28
8999 measured reflections	

### Refinement

**Table:**

Refinement on *F*^2^	Primary atom site location: structure-invariant direct methods
Least-squares matrix: full	Secondary atom site location: difference Fourier map
*R*[*F*^2^ > 2σ(*F*^2^)] = 0.046	Hydrogen site location: difference Fourier map
*wR*(*F*^2^) = 0.139	H-atom parameters constrained
*S* = 1.10	*w* = 1/[σ^2^(*F*_o_^2^) + (0.0886*P*)^2^] where *P* = (*F*_o_^2^ + 2*F*_c_^2^)/3
3280 reflections	(Δ/σ)_max_ = 0.001
152 parameters	Δρ_max_ = 1.28 e Å^−^^3^
0 restraints	Δρ_min_ = −0.59 e Å^−^^3^

### Special details

**Table:**

Experimental. Empirical absorption correction using spherical harmonics, implemented in SCALE3 ABSPACK scaling algorithm.
Geometry. All e.s.d.'s (except the e.s.d. in the dihedral angle between two l.s. planes) are estimated using the full covariance matrix. The cell e.s.d.'s are taken into account individually in the estimation of e.s.d.'s in distances, angles and torsion angles; correlations between e.s.d.'s in cell parameters are only used when they are defined by crystal symmetry. An approximate (isotropic) treatment of cell e.s.d.'s is used for estimating e.s.d.'s involving l.s. planes.
Refinement. Refinement of *F*^2^ against ALL reflections. The weighted *R*-factor *wR* and goodness of fit *S* are based on *F*^2^, conventional *R*-factors *R* are based on *F*, with *F* set to zero for negative *F*^2^. The threshold expression of *F*^2^ > σ(*F*^2^) is used only for calculating *R*-factors(gt) *etc*. and is not relevant to the choice of reflections for refinement. *R*-factors based on *F*^2^ are statistically about twice as large as those based on *F*, and *R*- factors based on ALL data will be even larger.

### Fractional atomic coordinates and isotropic or equivalent isotropic displacement parameters (Å^2^)

**Table:**

	*x*	*y*	*z*	*U*_iso_*/*U*_eq_	
Ca1	0.0000	0.11798 (9)	0.7500	0.02738 (15)	
P1	−0.08497 (3)	0.40618 (9)	0.59729 (3)	0.03320 (16)	
S1	0.11429 (2)	0.61596 (8)	0.80223 (2)	0.02767 (15)	
N1	0.10168 (11)	0.6246 (3)	0.86566 (10)	0.0398 (4)	
O1	0.08963 (8)	0.4015 (2)	0.76256 (7)	0.0361 (3)	
O2	0.08568 (8)	−0.1711 (3)	0.76345 (8)	0.0407 (4)	
O3	−0.03860 (8)	0.2157 (3)	0.63913 (7)	0.0386 (3)	
O4	−0.05382 (10)	0.5213 (3)	0.55067 (8)	0.0476 (4)	
O5	−0.15878 (8)	0.3097 (3)	0.54554 (8)	0.0506 (4)	
C1	0.20763 (10)	0.6308 (3)	0.82721 (10)	0.0299 (4)	
C2	0.24528 (12)	0.8183 (4)	0.86721 (13)	0.0457 (5)	
H2	0.2221	0.9313	0.8812	0.055*	
C3	0.31817 (13)	0.8330 (5)	0.88576 (15)	0.0565 (7)	
H3	0.3441	0.9571	0.9126	0.068*	
C4	0.35270 (13)	0.6668 (5)	0.86510 (14)	0.0518 (6)	
H4	0.4015	0.6799	0.8774	0.062*	
C5	0.31461 (12)	0.4795 (5)	0.82586 (12)	0.0488 (6)	
H5	0.3379	0.3667	0.8119	0.059*	
C6	0.24168 (12)	0.4602 (4)	0.80730 (11)	0.0397 (5)	
H6	0.2161	0.3333	0.7817	0.048*	
C7	0.00791 (16)	0.6710 (5)	0.57917 (17)	0.0597 (7)	
H7C	−0.0048	0.8154	0.5940	0.090*	
H7B	0.0251	0.7052	0.5462	0.090*	
H7A	0.0446	0.5927	0.6164	0.090*	
C8	−0.16248 (17)	0.0968 (5)	0.50864 (15)	0.0606 (8)	
H8C	−0.2108	0.0719	0.4763	0.091*	
H8B	−0.1472	−0.0345	0.5388	0.091*	
H8A	−0.1319	0.1115	0.4863	0.091*	

### Atomic displacement parameters (Å^2^)

**Table:**

	*U*^11^	*U*^22^	*U*^33^	*U*^12^	*U*^13^	*U*^23^
Ca1	0.0234 (2)	0.0265 (3)	0.0295 (3)	0.000	0.0087 (2)	0.000
P1	0.0313 (3)	0.0385 (3)	0.0308 (3)	0.00411 (19)	0.0142 (2)	0.0013 (2)
S1	0.0241 (2)	0.0263 (2)	0.0343 (3)	−0.00034 (15)	0.01395 (19)	0.00146 (16)
N1	0.0460 (10)	0.0390 (10)	0.0458 (11)	−0.0092 (8)	0.0305 (9)	−0.0062 (8)
O1	0.0359 (7)	0.0375 (8)	0.0371 (8)	−0.0088 (6)	0.0176 (6)	−0.0062 (6)
O2	0.0320 (7)	0.0384 (8)	0.0543 (9)	0.0098 (6)	0.0210 (7)	0.0140 (7)
O3	0.0413 (8)	0.0406 (8)	0.0311 (7)	0.0107 (6)	0.0126 (6)	−0.0017 (6)
O4	0.0515 (9)	0.0581 (10)	0.0415 (8)	0.0041 (8)	0.0280 (7)	0.0036 (8)
O5	0.0340 (8)	0.0555 (10)	0.0503 (10)	0.0032 (7)	0.0065 (7)	−0.0052 (8)
C1	0.0257 (8)	0.0337 (9)	0.0300 (9)	0.0009 (7)	0.0115 (7)	0.0019 (7)
C2	0.0365 (11)	0.0409 (11)	0.0571 (14)	−0.0054 (9)	0.0174 (10)	−0.0132 (10)
C3	0.0370 (11)	0.0575 (15)	0.0651 (16)	−0.0168 (11)	0.0124 (11)	−0.0087 (13)
C4	0.0285 (10)	0.0703 (16)	0.0541 (14)	0.0023 (11)	0.0153 (10)	0.0073 (13)
C5	0.0360 (11)	0.0663 (16)	0.0472 (13)	0.0121 (11)	0.0206 (10)	0.0021 (12)
C6	0.0334 (10)	0.0450 (11)	0.0389 (11)	0.0060 (9)	0.0136 (8)	−0.0042 (9)
C7	0.0610 (16)	0.0568 (15)	0.082 (2)	−0.0038 (13)	0.0498 (16)	−0.0032 (15)
C8	0.0541 (15)	0.0649 (18)	0.0486 (15)	−0.0044 (12)	0.0084 (12)	−0.0119 (12)

### Geometric parameters (Å, °)

**Table:**

Ca1—O3^i^	2.3055 (15)	O5—C8	1.443 (3)
Ca1—O3	2.3055 (15)	C1—C6	1.377 (3)
Ca1—O2^i^	2.3392 (15)	C1—C2	1.391 (3)
Ca1—O2	2.3392 (15)	C2—C3	1.386 (3)
Ca1—O1	2.3802 (14)	C2—H2	0.9300
Ca1—O1^i^	2.3802 (14)	C3—C4	1.375 (4)
Ca1—P1^i^	3.4863 (6)	C3—H3	0.9300
Ca1—P1	3.4863 (6)	C4—C5	1.387 (4)
P1—O3	1.4801 (15)	C4—H4	0.9300
P1—O5	1.5664 (17)	C5—C6	1.389 (3)
P1—O4	1.5748 (17)	C5—H5	0.9300
P1—N1^i^	1.6043 (19)	C6—H6	0.9300
S1—O2^ii^	1.4547 (15)	C7—H7C	0.9600
S1—O1	1.4603 (14)	C7—H7B	0.9600
S1—N1	1.5370 (19)	C7—H7A	0.9600
S1—C1	1.7695 (19)	C8—H8C	0.9600
N1—P1^i^	1.6043 (19)	C8—H8B	0.9600
O2—S1^iii^	1.4547 (15)	C8—H8A	0.9600
O4—C7	1.437 (4)		
			
O3^i^—Ca1—O3	152.17 (8)	O1—S1—N1	115.20 (9)
O3^i^—Ca1—O2^i^	101.74 (5)	O2^ii^—S1—C1	105.07 (9)
O3—Ca1—O2^i^	97.69 (6)	O1—S1—C1	106.42 (9)
O3^i^—Ca1—O2	97.69 (6)	N1—S1—C1	107.50 (11)
O3—Ca1—O2	101.74 (5)	S1—N1—P1^i^	127.01 (12)
O2^i^—Ca1—O2	90.94 (8)	S1—O1—Ca1	133.51 (9)
O3^i^—Ca1—O1	79.32 (5)	S1^iii^—O2—Ca1	138.86 (9)
O3—Ca1—O1	81.97 (6)	P1—O3—Ca1	132.93 (9)
O2^i^—Ca1—O1	177.86 (5)	C7—O4—P1	119.51 (17)
O2—Ca1—O1	87.07 (5)	C8—O5—P1	120.50 (17)
O3^i^—Ca1—O1^i^	81.97 (6)	C6—C1—C2	121.22 (19)
O3—Ca1—O1^i^	79.32 (5)	C6—C1—S1	120.38 (15)
O2^i^—Ca1—O1^i^	87.07 (5)	C2—C1—S1	118.40 (15)
O2—Ca1—O1^i^	177.86 (5)	C3—C2—C1	118.5 (2)
O1—Ca1—O1^i^	94.93 (8)	C1—C2—H2	120.6
O3^i^—Ca1—P1^i^	18.11 (4)	C3—C2—H2	120.6
O3—Ca1—P1^i^	136.46 (4)	C4—C3—C2	121.0 (2)
O2^i^—Ca1—P1^i^	119.57 (4)	C4—C3—H3	119.7
O2—Ca1—P1^i^	99.47 (4)	C2—C3—H3	119.7
O1—Ca1—P1^i^	61.60 (4)	C3—C4—C5	119.9 (2)
O1^i^—Ca1—P1^i^	80.87 (4)	C3—C4—H4	120.0
O3^i^—Ca1—P1	136.46 (4)	C5—C4—H4	120.0
O3—Ca1—P1	18.11 (4)	C4—C5—C6	120.1 (2)
O2^i^—Ca1—P1	99.47 (4)	C4—C5—H5	119.9
O2—Ca1—P1	119.57 (4)	C6—C5—H5	119.9
O1—Ca1—P1	80.87 (4)	C1—C6—C5	119.3 (2)
O1^i^—Ca1—P1	61.60 (4)	C1—C6—H6	120.4
P1^i^—Ca1—P1	124.04 (2)	C5—C6—H6	120.4
O3—P1—O5	111.87 (10)	O4—C7—H7C	109.5
O3—P1—O4	112.15 (10)	O4—C7—H7B	109.5
O5—P1—O4	102.01 (10)	H7C—C7—H7B	109.5
O3—P1—N1^i^	117.84 (9)	O4—C7—H7A	109.5
O5—P1—N1^i^	106.83 (10)	H7C—C7—H7A	109.5
O4—P1—N1^i^	104.71 (10)	H7B—C7—H7A	109.5
O3—P1—Ca1	28.96 (6)	O5—C8—H8C	109.5
O5—P1—Ca1	118.53 (8)	O5—C8—H8B	109.5
O4—P1—Ca1	130.87 (7)	H8C—C8—H8B	109.5
N1^i^—P1—Ca1	89.68 (7)	O5—C8—H8A	109.5
O2^ii^—S1—O1	112.75 (9)	H8C—C8—H8A	109.5
O2^ii^—S1—N1	109.23 (10)	H8B—C8—H8A	109.5
			
O3^i^—Ca1—P1—O3	155.86 (11)	P1—Ca1—O1—S1	106.66 (12)
O2^i^—Ca1—P1—O3	−85.79 (14)	O3^i^—Ca1—O2—S1^iii^	52.16 (15)
O2—Ca1—P1—O3	10.74 (14)	O3—Ca1—O2—S1^iii^	−147.87 (15)
O1—Ca1—P1—O3	92.07 (14)	O2^i^—Ca1—O2—S1^iii^	−49.81 (12)
O1^i^—Ca1—P1—O3	−167.21 (14)	O1—Ca1—O2—S1^iii^	130.96 (15)
P1^i^—Ca1—P1—O3	138.45 (14)	P1^i^—Ca1—O2—S1^iii^	70.39 (15)
O3^i^—Ca1—P1—O5	−120.27 (10)	P1—Ca1—O2—S1^iii^	−151.26 (13)
O3—Ca1—P1—O5	83.87 (16)	O5—P1—O3—Ca1	−109.73 (13)
O2^i^—Ca1—P1—O5	−1.92 (9)	O4—P1—O3—Ca1	136.34 (13)
O2—Ca1—P1—O5	94.62 (9)	N1^i^—P1—O3—Ca1	14.66 (17)
O1—Ca1—P1—O5	175.94 (9)	O3^i^—Ca1—O3—P1	−37.12 (12)
O1^i^—Ca1—P1—O5	−83.34 (9)	O2^i^—Ca1—O3—P1	96.95 (13)
P1^i^—Ca1—P1—O5	−137.68 (8)	O2—Ca1—O3—P1	−170.47 (13)
O3^i^—Ca1—P1—O4	98.13 (11)	O1—Ca1—O3—P1	−85.18 (13)
O3—Ca1—P1—O4	−57.74 (17)	O1^i^—Ca1—O3—P1	11.43 (13)
O2^i^—Ca1—P1—O4	−143.52 (10)	P1^i^—Ca1—O3—P1	−52.92 (16)
O2—Ca1—P1—O4	−46.99 (11)	O3—P1—O4—C7	−72.2 (2)
O1—Ca1—P1—O4	34.34 (10)	O5—P1—O4—C7	167.92 (19)
O1^i^—Ca1—P1—O4	135.05 (11)	N1^i^—P1—O4—C7	56.7 (2)
P1^i^—Ca1—P1—O4	80.71 (10)	Ca1—P1—O4—C7	−46.0 (2)
O3^i^—Ca1—P1—N1^i^	−11.21 (9)	O3—P1—O5—C8	−43.3 (2)
O3—Ca1—P1—N1^i^	−167.07 (15)	O4—P1—O5—C8	76.7 (2)
O2^i^—Ca1—P1—N1^i^	107.14 (8)	N1^i^—P1—O5—C8	−173.7 (2)
O2—Ca1—P1—N1^i^	−156.32 (9)	Ca1—P1—O5—C8	−74.6 (2)
O1—Ca1—P1—N1^i^	−75.00 (8)	O2^ii^—S1—C1—C6	−118.55 (18)
O1^i^—Ca1—P1—N1^i^	25.72 (9)	O1—S1—C1—C6	1.2 (2)
P1^i^—Ca1—P1—N1^i^	−28.62 (7)	N1—S1—C1—C6	125.18 (18)
O2^ii^—S1—N1—P1^i^	145.87 (15)	O2^ii^—S1—C1—C2	61.4 (2)
O1—S1—N1—P1^i^	17.8 (2)	O1—S1—C1—C2	−178.77 (18)
C1—S1—N1—P1^i^	−100.63 (16)	N1—S1—C1—C2	−54.8 (2)
O2^ii^—S1—O1—Ca1	−100.86 (13)	C6—C1—C2—C3	1.3 (4)
N1—S1—O1—Ca1	25.45 (16)	S1—C1—C2—C3	−178.7 (2)
C1—S1—O1—Ca1	144.47 (12)	C1—C2—C3—C4	0.2 (4)
O3^i^—Ca1—O1—S1	−34.37 (12)	C2—C3—C4—C5	−0.9 (5)
O3—Ca1—O1—S1	124.94 (13)	C3—C4—C5—C6	0.2 (4)
O2—Ca1—O1—S1	−132.76 (13)	C2—C1—C6—C5	−1.9 (3)
O1^i^—Ca1—O1—S1	46.48 (10)	S1—C1—C6—C5	178.06 (18)
P1^i^—Ca1—O1—S1	−30.35 (10)	C4—C5—C6—C1	1.2 (4)

Symmetry codes: (i) −*x*, *y*, −*z*+3/2; (ii) *x*, *y*+1, *z*; (iii) *x*, *y*−1, *z*.
